# Accuracy and time efficiency of a novel deep learning algorithm for Intracranial Hemorrhage detection in CT Scans

**DOI:** 10.1007/s11547-024-01867-y

**Published:** 2024-08-09

**Authors:** Tommaso D’Angelo, Giuseppe M. Bucolo, Tarek Kamareddine, Ibrahim Yel, Vitali Koch, Leon D. Gruenewald, Simon Martin, Leona S. Alizadeh, Silvio Mazziotti, Alfredo Blandino, Thomas J. Vogl, Christian Booz

**Affiliations:** 1 Diagnostic and Interventional Radiology Unit, BIOMORF Department, University of Messina, Messina, Italy; 2 Department of Radiology and Nuclear Medicine, Erasmus MC, 3015 GD Rotterdam, The Netherlands; 3 Division of Experimental Imaging, Department of Diagnostic and Interventional Radiology, University Hospital Frankfurt, Frankfurt Am Main, Germany; 4 Department of Diagnostic and Interventional Radiology, University Hospital Frankfurt, Frankfurt Am Main, Germany; 5 Department of Diagnostic and Interventional Radiology and Neuroradiology, Bundeswehr Central Hospital Koblenz, Koblenz, Germany

**Keywords:** Intracranial hemorrhage, Tomography, X-ray computed, Artificial intelligence, Brain injuries, Traumatic, Diagnosis, Computer-assisted

## Abstract

**Purpose:**

To evaluate a deep learning-based pipeline using a Dense-UNet architecture for the assessment of acute intracranial hemorrhage (ICH) on non-contrast computed tomography (NCCT) head scans after traumatic brain injury (TBI).

**Materials and methods:**

This retrospective study was conducted using a prototype algorithm that evaluated 502 NCCT head scans with ICH in context of TBI.

Four board-certified radiologists evaluated in consensus the CT scans to establish the standard of reference for hemorrhage presence and type of ICH. Consequently, all CT scans were independently analyzed by the algorithm and a board-certified radiologist to assess the presence and type of ICH. Additionally, the time to diagnosis was measured for both methods.

**Results:**

A total of 405/502 patients presented ICH classified in the following types: intraparenchymal (*n* = 172); intraventricular (*n* = 26); subarachnoid (*n* = 163); subdural (*n* = 178); and epidural (*n* = 15). The algorithm showed high diagnostic accuracy (91.24%) for the assessment of ICH with a sensitivity of 90.37% and specificity of 94.85%. To distinguish the different ICH types, the algorithm had a sensitivity of 93.47% and a specificity of 99.79%, with an accuracy of 98.54%. To detect midline shift, the algorithm had a sensitivity of 100%. In terms of processing time, the algorithm was significantly faster compared to the radiologist’s time to first diagnosis (15.37 ± 1.85 vs 277 ± 14 s, *p* < 0.001).

**Conclusion:**

A novel deep learning algorithm can provide high diagnostic accuracy for the identification and classification of ICH from unenhanced CT scans, combined with short processing times. This has the potential to assist and improve radiologists’ ICH assessment in NCCT scans, especially in emergency scenarios, when time efficiency is needed.

## Introduction

Traumatic brain injury (TBI) is a severe neurologic emergency with high morbidity and mortality. The deterioration of patient's condition begins within the first few hours after the onset of trauma, and delayed diagnosis can reduce patient’s recovery [1].

Intracranial hemorrhage (ICH) constitutes a severe consequence of TBI, categorized into four distinct types based on anatomical location: epidural hemorrhage (EDH), subdural hemorrhage (SDH), intraparenchymal hemorrhage (IPH), and subarachnoid hemorrhage (SAH). Complications arise from elevated intracerebral pressure due to hemorrhage itself, peri-lesional edema, or hydrocephalus resulting from obstruction of cerebrospinal fluid. A pivotal radiological indicator of mass effect is the midline shift (MSH), commonly observed in patients with ICH and strongly correlated with increased morbidity and mortality. Non-contrast computed tomography (NCCT) imaging of the head is the modality of choice for the evaluation of TBI in emergency setting, due to its ease of execution, broad availability, and cheap cost. It actually serves as the standard of care in the majority of centers for diagnosis of head injuries and acute strokes [2]. Its efficacy as blood detector is notable, as it can clearly show the hyperdensity of acute bleeding compared to the normal cerebral parenchyma. Additionally, the CT scan provides important details about midline shift, serving as an indicator of the severity of patient’s condition.

The treatment approach for TBI depends on its severity and patient's clinical condition. The physician usually performs clinical, neurological, and imaging assessment to determine the impact of a head injury. ICH must be accurately and rapidly detected if present, to avoid poor clinical outcome. In case of ICH, prompt therapeutic approaches such as hemostasis, blood pressure control, invasive surgical procedures, and intracranial pressure management may all represent lifesaving procedures [3, 4].

Clinical processes have been implemented in various areas to reduce the time to diagnosis and time to treatment [5]. In this setting, a quick automated scan evaluation for detection of ICH may play a pivotal role. Large imaging datasets are becoming easily accessible as a result of the usual storage of clinical data, and they can potentially provide a rich source for scientific testing and for the development of deep learning algorithms. These algorithms constitute the basis of artificial intelligence (AI) and have been doing constant advancements in different medical fields. In particular, AI has shown its potential to evaluate CT scans in a few seconds by means of machine learning (ML) [6].

As imaging experts, radiologists are asked to be responsible and contributors for the acknowledgment of AI applications, by understanding and monitoring their effectiveness [7].

In this context, our study aims to investigate the processing time and the diagnostic performance of a novel deep neural network algorithm for the detection of ICH and brain midline shift (MLS).

## Materials and methods

The need for informed consent has been waived by the institutional review board (*n*. 19–236). Anonymized NCCT head scans from patients entering our Central Emergency department were retrospectively retrieved from our institutional database.

### Study design

In this retrospective study, consecutive scans of patients who underwent NCCT scan after TBI between January 2013 and December 2019 were examined. All NCCT scans were performed on two different CT scanners (CT Somatom Force and CT Somatom Definition AS, Siemens Healthineers, Forchheim, Germany). All examinations were performed using the institutional standardized imaging protocol for NCCT head scans. Two image datasets were reconstructed with a slice thickness of 1 mm and 4 mm by means of a dedicated iterative noise reduction algorithm (ADMIRE).

**Fig. 1:**
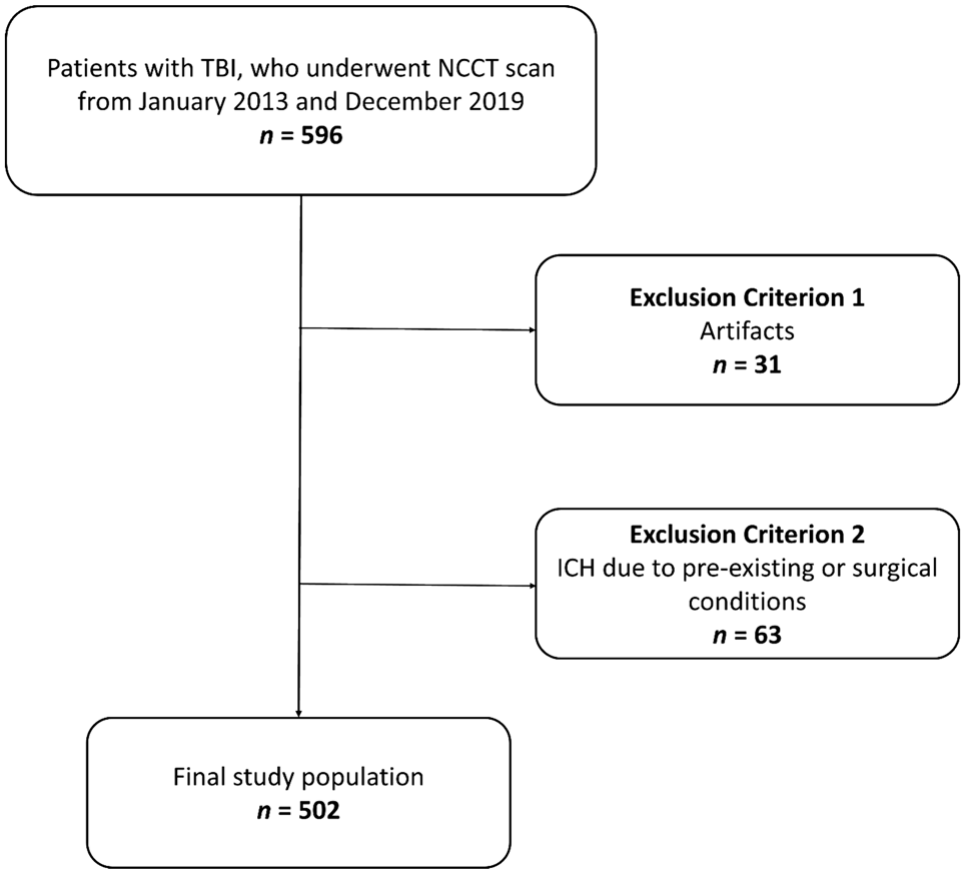
Flowchart showing patient inclusion and exclusion criteria for this study. TBI: trauma brain injury; NCCT: non-contrast computed tomography; and ICH: intracranial hemorrhage

The exclusion criteria were the presence of artifacts (*n*: 31), cerebral hemorrhage due to preexisting conditions (*n*: 63), such as brain metastases, and surgical treatment (i.e., craniotomy). The presence of exclusion criteria was verified by a board-certified radiologist with 8 years of experience in CT imaging (CB). Figure 1 shows the patient selection flowchart.

The presence, the type and the location of ICH and the presence of MLS were checked and reported in consensus sessions by four board-certified radiologists (TJV, CB, SM, LG), respectively, with 32, 9, 8, and 7 years of experience in CT imaging, in order to establish the reference standard. Notably, they were deliberately kept unaware of the patients' clinical status to maintain the integrity of the reference standard.

Subsequently, a board-certified radiologist (TD) with 8 years of experience in CT imaging was asked to establish the presence and type of ICH in all CT scans, as well as the presence of MLS. In addition, the radiologist’s reading times were recorded.

Consequently, anonymized DICOM files with 4 mm reconstructions were exported and forwarded to Siemens Healthineers’ headquarter for the algorithm analysis. The algorithm only provided information on the presence or absence of bleeding, without a further analysis of the subtype of bleeding. The false-positive cases were reviewed by the radiologist, who categorized the misidentified hemorrhage according to its anatomical location. 

All radiologists participating in this study were members of the same cohesive team and department.

### Deep neural network algorithm

The algorithm evaluated in this study was developed by Siemens Healthineers (Forchheim, Germany) [8]. A deep learning method for volumetric segmentation of biomedical images, known as 3D U-Net, is the basis of this approach. The algorithm can learn from sparsely annotated bidimensional images, resulting in a 3D segmentation, and is able to analyze NCCT scans for ICH [9, 10]. Zheng et al. [11] improved upon this algorithm by developing a densely connected convolutional network called Dense-UNet, which introduces additional concatenation layers between each pair of convolutional layers. This design allows each layer to receive feature maps from all pending layers as input and pass its feature maps to all subsequent layers. Consequently, segmentation quality is enhanced without the need for larger datasets. Siemens Healthineers developed an AI pipeline based on the Dense-UNet architecture for automated NCCT processing.

During initialization, the layout of the brain is adjusted by spotting landmarks within the anatomy. The pipeline starts by detecting five key anatomical landmarks in the brain (Bregma, Crista Galli, External Occipital Protuberance, Left and Right Orbital Bones) to estimate its orientation and reduce head position variability [12]. Using an image-to-image convolutional network trained with deep supervision and adversarial perturbations, the brain region is extracted, excluding robust features like the skull that could mislead the detection process [13]. The respective algorithm is based on deep reinforcement learning at multiple scales [12]. By training an image-to-image segmentation networking with deep monitoring and antagonistic disruptions, the brain extraction and elimination of prominent characteristics, for example, bone structures, are achieved [14]. Following this initial phase of analysis, a set of deep compact neural networks that retrieve characteristics from both axial and coronal planes are used to detect the occurrence or non-occurrence of ICH at the case scale [8]. The network is trained full-length with voxel-level monitoring on the applicable hemorrhage masking and mark monitoring on general occurrence or non-occurrence.

In this study, we evaluated the capability of this novel algorithm to assess the presence of ICH and the potential presence of MLS.

### Measurement of latency time until reporting of ICH

The time interval between image reconstruction, transfer to the institutional picture archiving and communication system (PACS), and first radiologist’s result was used to calculate the result latency time of the standard procedure.

Similarly, the time between image transfer to the deep learning workstation and first notification of detected ICH (notification within PACS stating “bleeding detected/no bleeding detected”) was considered the result latency time of the novel procedure using the AI algorithm.

### Statistical analysis

Statistical analysis was performed using dedicated statistical software (Medcalc; MedCalc Software, Ostend, Belgium).

In our comprehensive evaluation of the AI algorithm's performance, we analyzed various metrics, including specificity, sensitivity, positive and negative predictive values. Additionally, the assessment of the overall accuracy was evaluated besides positive and negative likelihood ratios. Moreover, we systematically recorded the processing time for both AI software and radiologists, comparing them using unpaired t test.

The statistically significant difference was indicated by a P value less than 0.05.

## Results

A total of 502 patients (mean age: 55 ± 12 years; range 23–95 years), comprising 274 males (54.58%; mean age of 53 ± 11; range 27–89), and 228 females (45.42%; mean age: 57 ± 13; range 23–95), who underwent NCCT scan for clinical suspicion of ICH were analyzed.

**Fig. 2:**
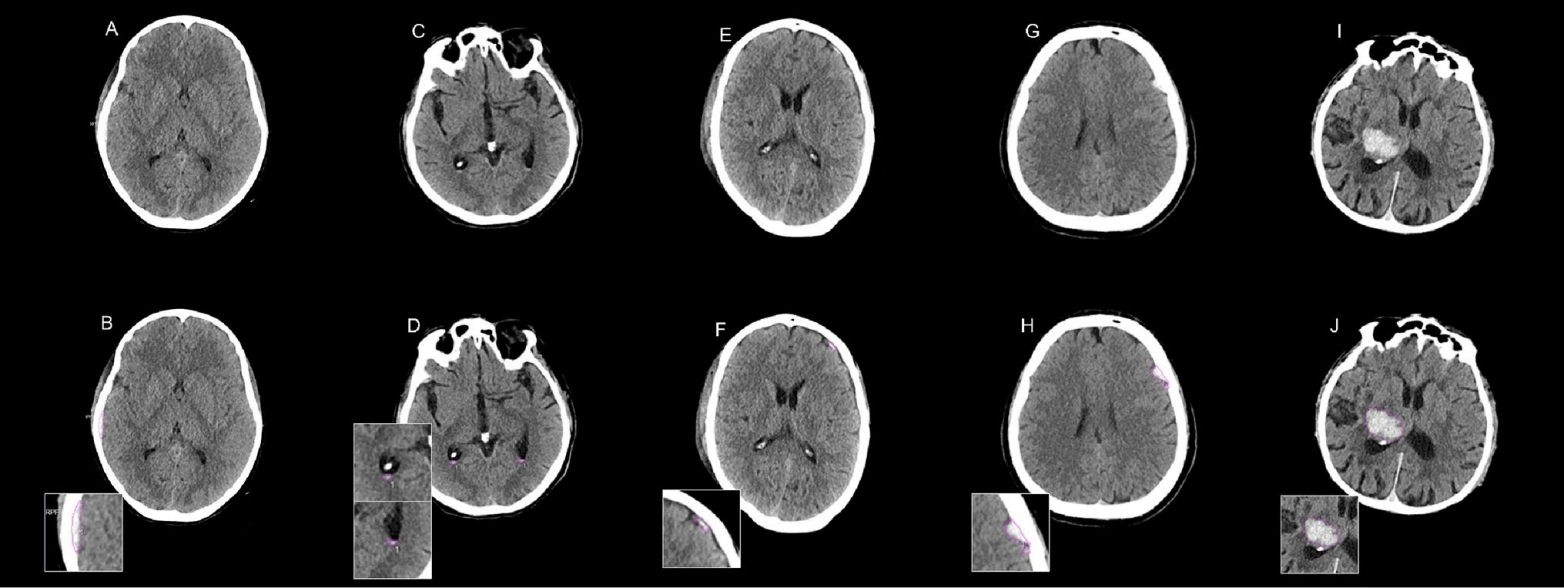
Representative images of automated detection of acute intracranial hemorrhage in acute traumatic brain injury (TBI). Examples include subdural hemorrhage (**A**, **B**), symmetrical intraventricular hemorrhage (**C**, **D**), subarachnoid hemorrhage (**E**, **F**), intraparenchymal hemorrhage (**G**, **H**), and epidural hemorrhage (**I**, **J**). The bleeding is shown as hyperdense areas in different regions of the brain with volumes ranging from 0.632 to 16.119 ml

Within this cohort, a total of 405 cases were positive for ICH according to our reference standard, resulting in a prevalence rate of 81%. The ICH-positive cases were further classified by radiologists into the following subtypes: intraparenchymal hemorrhage (IPH), accounting for 172 cases (42%), intraventricular hemorrhage (IVH), for 26 cases (6%), subarachnoid hemorrhage (SAH), for 163 cases (40%), subdural hemorrhage (SDH), for 178 cases (44%), and epidural hemorrhage (EDH), for 15 cases (4%) (Fig. 2). Notably, a subset of 115 ICH-positive cases (28%) concomitantly presented multiple hemorrhage subtypes. Additionally, a total of 43 patients (11%) exhibited MLS due to hematoma pressure and swelling.

The algorithm identified the presence of ICH in 366 patients, yielding an overall sensitivity of 90.37% (95%-CI: 87.11, 92.88), specificity of 94.85% (95%-CI: 88.50, 97.78), and accuracy of 91.24% (95%-CI: 88.44, 93.41). In total, there were 5 false-positive cases, as shown in Table 1. The evaluated positive and negative predictive values were 98.65% (95%-CI: 96.88, 99.42) and 70.23% (95%-CI: 61.91, 77.39), respectively.

**Table 1:** Summary of the performance values of the AI for the detection of intracranial hemorrhage

	Reference standard			AI Model								
	Total	Positives	Negatives	TP	TN	FP	FN	Sensitivity	Specificity	Accuracy	PPV	NPV
All Cases	502	405	97	366	92	5	39	90.37 (87.11, 92.88)	94.85 (88.50, 97.78)	91.24 (88.44, 93.41)	98.65 (96.88, 99.42)	70.23 (61.91, 77.39)

Data in brackets denote 95% confidence interval. Sensitivity, specificity, accuracy *PPV* (positive predictive value), and *NPV* (negative predictive value) are expressed in percentage (%)*TP* true positive, *TN* true negative, *FP* false positive, *FN* false negative

**Fig. 3:**
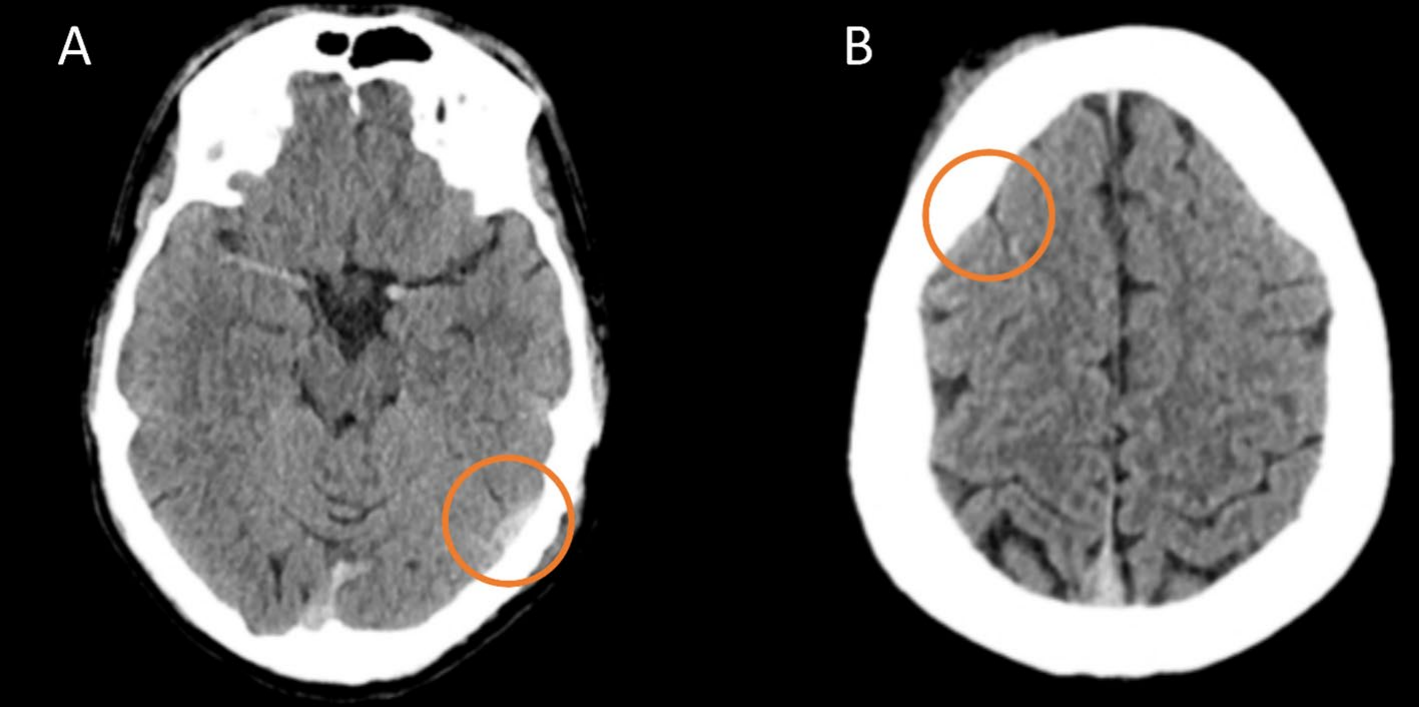
False-negative predictions by the AI model: **A** Missed subdural hemorrhage in the temporal lobe of the left hemisphere is marked with orange circle. **B** Missed subarachnoid hemorrhage located the frontal lobe of the right hemisphere is marked with orange circle

Moreover, the performance of the AI model by ICH subtype showed that the algorithm achieved an accuracy of 100% for IVH assessment (95%-CI: 99.27, 100), while for IPH detection the accuracy was the lowest (96.61%; 95%-CI: 94.63, 98.02), followed by SDH (97.41%; 95%-CI: 95.61, 98.61) and SAH (97.61%; 95%-CI: 95.86, 98.76) (Fig. 3). In addition, the AI model had an accuracy of 100% (95%-CI: 99.27, 100) for the identification of MLS. Table 2 provides a comprehensive overview of the ICH subtypes occurrence, together with the AI model performance per-subtype.

**Table 2:** ICH subtypes occurrence with AI model performance per-subtype

	Total	TP	TN	FP	FN	Sensitivity	Specificity	Accuracy	PPV	NPV
Cases with IPH	502	158	327	3	14	91.86 (86.72, 95.48)	99.09 (97.37, 99.81)	96.61 (94.63, 98.02)	98.14 (94.46, 99.39)	95.89 (93.39, 97.47)
Cases with IVH	502	26	476	0	0	100.00 (86.77, 100.00)	100.00 (99.23, 100.00)	100.00 (99.27, 100.00)	100.00 (86.77, 100.00)	100.00 (99.23, 100.00)
Cases with SAH	502	151	339	0	12	92.64 (87.49, 96.14)	100.00 (98.92, 100.00)	97.61 (95.86, 98.76)	100.00 (97.59, 100.00)	96.58 (94.25, 97.99)
Cases with SDH	502	166	323	1	12	93.26 (88.52, 96.47)	99.69 (98.29, 99.99)	97.41 (95.61, 98.61)	99.40 (95.91, 99.91)	96.42 (93.97, 97.89)
Cases with EDH	502	14	486	1	1	93.33 (68.05, 99.83)	99.79 (98.86, 99.99)	99.60 (98.57, 99.95)	93.33 (66.29, 99.01)	99.79 (98.65, 99.97)
Cases with MLS	502	43	459	0	0	100.00 (91.78, 100.00)	100.00 (99.20, 100.00)	100.00 (99.27, 100.00)	100.00 (91.78, 100.00)	100.00 (99.20, 100.00)

Data in brackets denote 95% confidence interval. Sensitivity, specificity, accuracy, *PPV* (positive predictive value), and *NPV* (negative predictive value) are expressed in percentage (%) *EDH* Epidural hemorrhage, *FN* false negative, *FP* false positive, *IPH* intraparenchymal hemorrhage, *IVH* Intraventricular hemorrhage, *MLS* Midline shift, *SAH* Subarachnoid hemorrhage, *SDH* Subdural hemorrhage *TN* true negative, *TP* true positive

The algorithm processing time had an average value of 15.37 s, ranging from the fastest detection time of 11.86 s to the slowest of 18.69 s. In comparison with the radiologist’s detection time, the algorithm was significantly faster (15.37 ± 1.85 s vs 277 ± 14 s, *p* < 0.001).

## Discussion

This study evaluated a deep neural network algorithm prototype that may detect an intracranial hemorrhage based on NCCT head scans. Our results showed high diagnostic performance resulting in an overall accuracy of 91.24%, with sensitivity of 90.37%, specificity of 94.85%, and an average processing time of 15.37 s. The algorithm achieved accuracies of 97.41% and 96.61% for the two most prevalent ICH subtypes SDH and IPH in our cohort (prevalence of 44% and 42%, respectively).

Previous studies have also been conducted to assess the use of AI for the detection of ICH. Gruschwitz et al. [15] evaluated the performance of an AI algorithm using both a retrospective dataset of 872 CT scans and a prospective dataset of 100 CT scans. Their results demonstrated high sensitivity and specificity in detecting ICH, resulting in an overall accuracy of 91.0%, with a processing time of less than 30 s. Interestingly, when the software was used in conjunction with a human radiologist, the sensitivity raised to 100%. Moreover, Gibson et al. [8] evaluated 46,057 studies from 10 centers using an AI model, showing high performance with an accuracy of 97% (sensitivity: 92%; specificity: 93%) and 95% (sensitivity: 86%; specificity: 93%) for the internal and external centers, respectively. In addition, they evaluated the report turnaround time and found that this could be improved by an additional 25–27%.

According to the scientific literature, our results showed similar diagnostic performance parameters to different AI-algorithms currently available. Furthermore, we conducted a comparison between results obtained by the deep neural network algorithm and the ICH subtype determined through the radiologists’ assessment. Our findings revealed that the algorithm shows a sensitivity of 93.47% and a specificity of 99.79% in distinguishing ICH subtypes. Notably, the highest accuracy has been demonstrated in identifying IVH. This can be explained by the relatively stable location of IVH, since each cerebral hemisphere contains the lateral ventricle, which consists of a C-shaped cavity that exhibits lower density than normal brain parenchyma. Consequently, any occurrence of ICH within the ventricles may contribute to the good algorithm performance [16, 17]. Furthermore, hyperdense structures commonly found at this level, such as calcifications of the choroid plexuses, can be readily identified by the AI software owing to their stable location and symmetry.

**Fig. 4:**
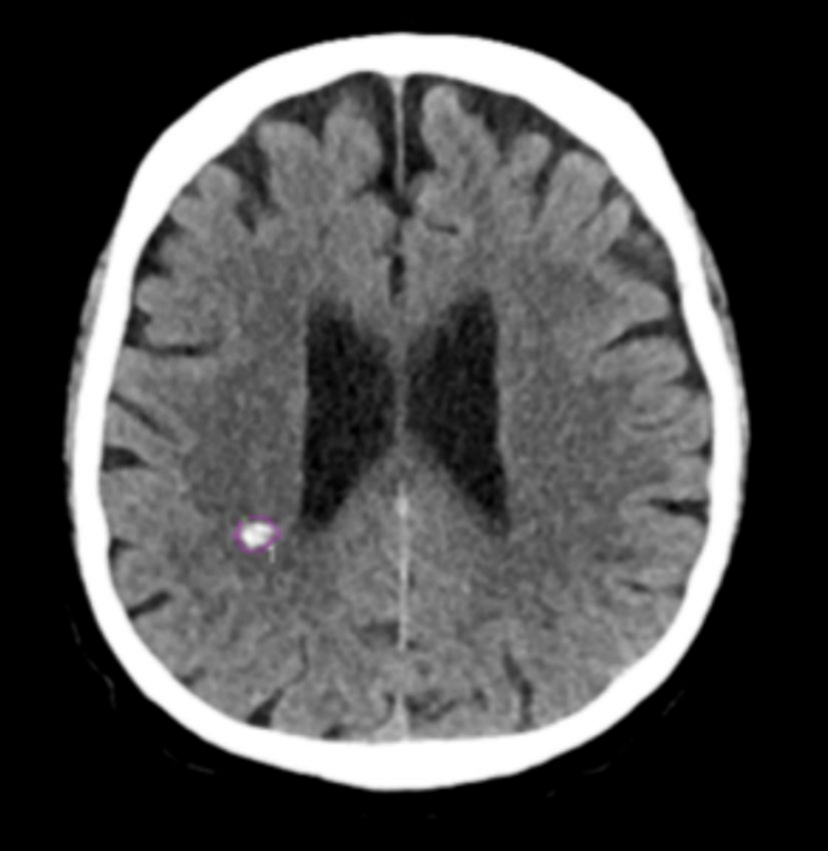
Example of a false-positive case that resembles a strong hyperdense calcification in the parietal lobe of the right hemisphere. The calcification in the right parietal lobe was interpreted falsely as intraparenchymal hemorrhage by the algorithm

On the other hand, the detection of IPH obtained the lowest accuracy. This result may depend on different factors that make IPH identification more challenging. One aspect is related to the random location of IPH, which may potentially occur anywhere within the brain parenchyma. Moreover, the variable size of IPH may add to the complexity of its detection [18]. Another major aspect to consider is the presence of parenchymal calcifications. In fact, due to their high density on CT images, small calcifications may be misinterpreted as small hemorrhage (Fig. 4). This issue is not limited to IPH detection but also applies to the other types of ICH, and it represents one of the main challenges that can lead to false-positive findings.

Our results showed faster processing-time compared to previous studies, with a maximum time that is inferior to 20 s and a mean value of 15.37 s, which indicates a potential benefit of using this algorithm, compared to the other existing prototypes.

Nowadays, AI technologies can be used and adopted in medical practice and radiological routines, improving the efficiency of decision-making tasks and medical diagnosis [19]. Furthermore, technological advances in emergent AI systems may further automate and enhance the efficacy of decision-making tasks in clinical settings, as medical evaluation, especially considering that radiologists’ workload is continuously increasing [6, 20, 21].

In a study conducted by Chien et al. [22], the application of AI software, known as DeepCT, was assessed in hemorrhagic stroke. Their results substantiated that the integration of AI can effectively save costs, decrease ED stay, and accelerate patient workflow.

However, excessive workload can negatively impact the level of diagnosis and treatment that healthcare professionals can deliver [23]. The increasing number of scans to be evaluated often pushes radiologists to accelerate reporting, leading to a higher likelihood of inconsistencies in diagnostic interpretations. A previous study showed that halving the analysis time of radiologist analysis duration increases the incidence of inaccuracies by 16.6%, thereby compromising the efficacy and quality of patient’s treatment. Consequently, the integration of Al within the diagnostic workflow may alleviate the workload by performing routine tasks in shorter timeframes, allowing physicians to focus more on complex cases that require human assessment and improving the patient management [24].

Undoubtedly, a comprehensive clinical validation is mandatory before the integration of any AI-based application into actual clinical workflows. Moreover, the integration of AI alongside the expertise of the users is probably the key to exploit its value in clinical routine.

In a recent meta-analysis study [25], 29 studies were included to assess the diagnostic accuracy of ML algorithms in detecting ICH in NCCT scans. Their results demonstrated a good AI performance, with an aggregated sensitivity of 91.7% and specificity of 94.5%, suggesting a promising role in the detection of ICH.

In our study, the evaluation of this novel algorithm showed both high accuracy and high-speed processing, which may translate into support for clinicians to give quicker answers. In fact, AI other than helping for providing accurate diagnosis can also help prioritizing patients at risk in stressful scenarios such as emergencies, where multiple patients may simultaneously require a NCCT.

**Fig. 5:**
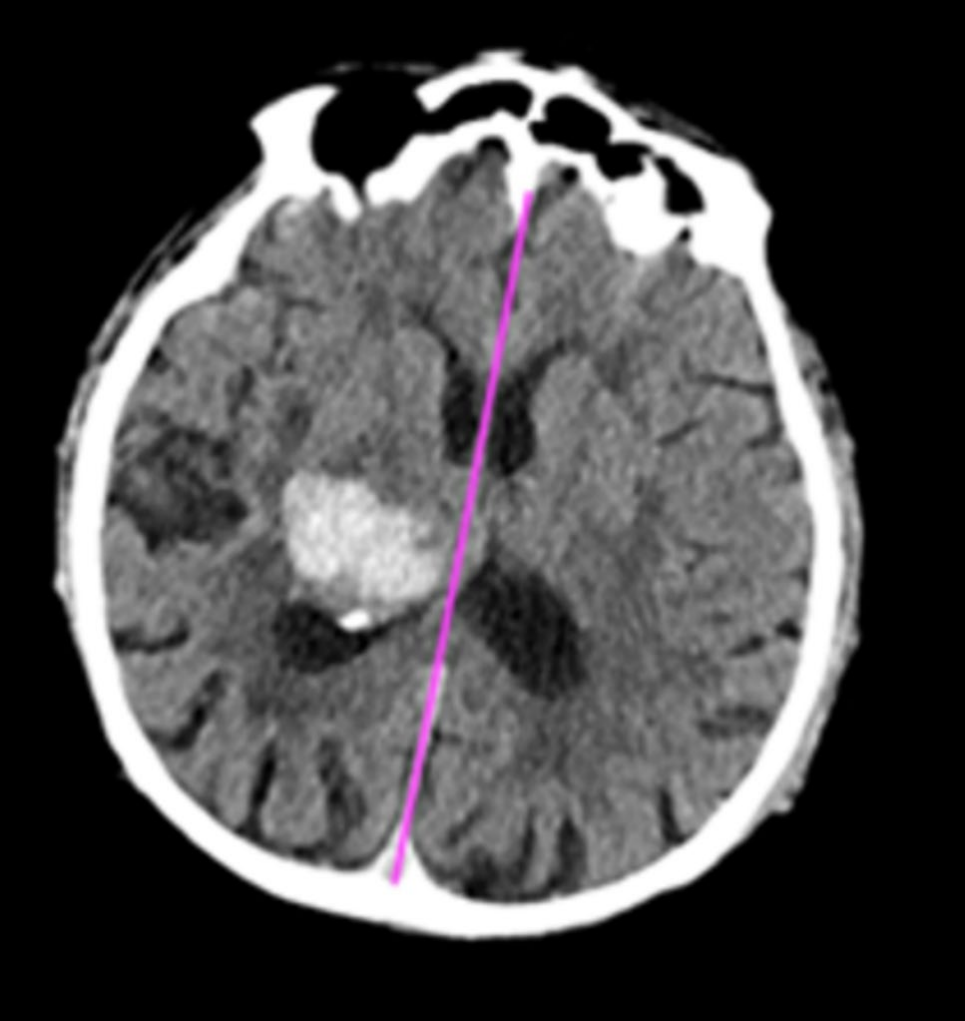
CT scan with the presence of a basal ganglia hemorrhage with related midline shift

Our findings showed that the deep neural network algorithm successfully detected ICH in all cases of MLS, which represents the typical scenario where a quick diagnosis is crucial due to the potential for rapid neurological deterioration and the need for urgent intervention (Fig. 5).

The integration of similar AI models with the radiologist's expertise may create a synergistic association that can maximize diagnosis accuracy, efficiency, and quality of medical imaging, with benefits for patients’ care and outcomes.

However, there are several limitations of this study that need to be addressed. Firstly, all CT scans have been performed in a single center and retrospectively analyzed, which may limit the application of our results to different contexts. Secondly, the case enrollment was highly selective, focusing only on traumatic hemorrhage assessment. In fact, patients with ICH related to treatment outcomes or other preexisting lesions were excluded from the analysis. Thus, we have not evaluated the assessment of ICH unrelated to trauma. Lastly, the deep neural network algorithm detected ICH from NCCT scans independently from patient’s clinical status or comorbidities. The degree of acuity of such findings may also be related to clinical or anamnestic data.

In conclusion, this pilot study demonstrated that a novel deep neural network algorithm yields high diagnostic performance in combination with time efficiency for the assessment of ICH and MLS in NCCT head scans in context of TBI. The algorithm has the potential to assist radiologists in clinical routine, thereby reducing the likelihood of missing ICH and optimizing reporting time. Additionally, its integration into clinical workflows holds the potential to ensure accurate and prioritized management, enabling timely and appropriate treatments.

## Data Availability

The full dataset will not be provided for publication at this moment due to further upcoming research projects related to department of University Hospital Frankfurt.

## Abbreviations

AI: Artificial intelligence
CI: Confidence interval
EDH: Epidural hemorrhage
ICH: Intracranial hemorrhage
IPH: Intraparenchymal hemorrhage
IVH: Intraventricular hemorrhage
ML: Machine learning
MLS: Midline shift
NCCT: Non-contrast computed tomography
SAH: Subarachnoid hemorrhage
SDH: Subdural hemorrhage
TBI: Traumatic brain injury
